# Contribution of Intracellular Calcium and pH in Ischemic Uncoupling of Cardiac Gap Junction Channels Formed of Connexins 43, 40, and 45: A Critical Function of C-Terminal Domain

**DOI:** 10.1371/journal.pone.0060506

**Published:** 2013-03-25

**Authors:** Giriraj Sahu, Amal Kanti Bera

**Affiliations:** Department of Biotechnology, Indian Institute of Technology Madras, Chennai, Tamil Nadu, India; National Institutes of Health, United States of America

## Abstract

Ischemia is known to inhibit gap junction (GJ) mediated intercellular communication. However the detail mechanisms of this inhibition are largely unknown. In the present study, we determined the vulnerability of different cardiac GJ channels formed of connexins (Cxs) 43, 40, and 45 to simulated ischemia, by creating oxygen glucose deprived (OGD) condition. 5 minutes of OGD decreased the junctional conductance (G_j_) of Cx43, Cx40 and Cx45 by 53±3%, 64±1% and 85±2% respectively. Reduction of G_j_ was prevented completely by restricting the change of both intracellular calcium ([Ca^2+^]_i_) and pH (pH_i_) with potassium phosphate buffer. Clamping of either [Ca^2+^]_i_ or pH_i_, through BAPTA (2 mM) or HEPES (80 mM) respectively, offered partial resistance to ischemic uncoupling. Anti-calmodulin antibody attenuated the uncoupling of Cx43 and Cx45 significantly but not of Cx40. Furthermore, OGD could reduce only 26±2% of G_j_ in C-terminus (CT) truncated Cx43 (Cx43-*Δ*257). Tethering CT of Cx43 to the CT-truncated Cx40 (Cx40-Δ249), and Cx45 (Cx45-Δ272) helped to resist OGD mediated uncoupling. Moreover, CT domain played a significant role in determining the junction current density and plaque diameter. Our results suggest; OGD mediated uncoupling of GJ channels is primarily due to elevated [Ca^2+^]_i_ and acidic pH_i_, though the latter contributes more. Among Cx43, Cx40 and Cx45, Cx43 is the most resistant to OGD while Cx45 is the most sensitive one. CT of Cx43 has major necessary elements for OGD induced uncoupling and it can complement CT of Cx40 and Cx45.

## Introduction

Gap junctions (GJs) participate in traffic of signalling molecules and propagation of electrical impulse between adjacent cells by forming intercellular channels. Six connexin (Cx) molecules assemble to form cell surface hemichannel, whereas GJs are formed by the docking of two hemichannels from the adjacent cells [1]. Till date, more than 20 Cxs have been reported in human [2]. Cxs have four transmembrane domains, two extracellular loops, one intracellular loop and cytoplasmic N and C-termini [1]. In mammalian heart, 3 major Cx isoforms i.e. Cx45, Cx43 and Cx40 are expressed. Expression of Cx43 predominates in all parts of the heart, whereas expression of Cx40 and Cx45 are compartmentalized [3], [4]. Overlapping expression pattern of Cxs raised the possibility of the formation of homomeric, heteromeric, homotypic and heterotypic junctions in heart [5]–[8]. Un-apposed hemichannels on the cell surface can function in certain physiological conditions, whereas GJs are constitutively active. The length of C-terminus (CT) varies among different Cx isoforms. CT plays a crucial role in the assembly, degradation and function of GJs. CT of Cx43 harbours multiple phosphorylation sites for several protein kinases [9], [10].

During ischemia, gap junctional communication is compromised which may cause cardiac arrhythmia [11]–[13]. Interestingly, hemichannels and GJs behave differently in response to metabolic inhibition or simulated ischemia. Metabolic inhibition induces the opening of hemichannels but reduces the gap junctional coupling [14], [15]. Uncoupling of GJs, limit the spread of ischemic damage [16]. The association of Cx43 in both ischemia-reperfusion injury and ischemic preconditioning, a mechanism by which repeated sub-lethal ischemia protects tissue from severe ischemia have been widely studied [17], [18]. Ischemia-induced dephosphorylation of Cx43 and its translocation to the intracellular pool plays a major contribution in the inhibition of junctional communication [12]. Ischemia lowers the intracellular pH (pH_i_) and increase cytoplasmic calcium ([Ca^2+^]_i_). Acidic pH_i_ and elevated [Ca^2+^]_i_ have been shown to disrupt cell-cell communication in many cells including cardiomyocytes [19], [20]. The inhibitory effect of [Ca^2+^]_i_ rise on junctional communication is possibly mediated through calmodulin [21]. Effect of intracellular acidification varies among different Cxs. Among the 3 cardiac Cxs, Cx45 and Cx40 showed highest and lowest pH-sensitivity, with Cx43 being intermediate [22]. CT of many Cxs dictates the pH sensitivity. Removal of CT impairs the pH gating of Cx43 and Cx40, but it had no effect on the pH sensitivity of Cx45. Further, a chimera of Cx40 having CT domain from Cx43 restored the pH sensitivity and vice versa [22].

Most of the studies relating to the ischemic uncoupling included ‘whole heart’ or ‘isolated cardiomyocytes’ where Cx45 and Cx40 containing GJs do exist, apart from Cx43 [12], [13], [23]. Therefore, the observed cumulative effect does not throw light on the susceptibility of individual Cxs to ischemia. Also, the molecular mechanism underlying ischemia mediated uncoupling of GJs is poorly understood. To study the effect of intracellular acidification on GJs, 100% CO_2_ have often been used, which hardly mimic ischemic condition. Most studies, related to the effect of pH_i_ or [Ca^2+^]_i_ on GJs studied individually in a non-ischemic milieu.

In the present study, we mimicked ischemia by creating oxygen glucose deprivation (OGD) and studied its effect on homomeric, homotypic GJ channels constituting of Cx45, Cx43 and Cx40, expressed in Neuro-2a (N2a) cells. The cumulative effect as well as individual contribution of pH_i_ drop and [Ca^2+^]_i_ rise on the uncoupling of different GJs were studied in OGD condition. We observed that Cx45 is more susceptible to OGD, compared to Cx43 and Cx40. OGD caused maximum inhibition of junctional conductance (G_j_) in Cx45 followed by Cx40 and Cx43. The role of CT in ischemic uncoupling was also studied by generating CT truncated mutants and swapping the CT between different Cxs.

## Materials and Methods

### Molecular biology

cDNA of mouse Cx45 and Cx40 was kindly provided by Prof. Klaus Willecke, Germany. Mouse eGFP tagged Cx43 (Cx43-eGFP) was provide by Prof. Feliksas F. Bukauskas, Albert Einstein College of Medicine, New York. Cx43, Cx45 and Cx40 were sub-cloned into pIRES2-DsRed or pIRES2-eGFP expression vector (Clontech, USA) by using standard molecular biology techniques. Cx45 was also sub-cloned into peGFPN1 vector (Clontech, USA) to express it as a fusion protein with eGFP. All the constructs were sequenced, followed by functional studies using patch clamp.

CT truncated Cx45, Cx43 and Cx40 were generated with the help of PCR based site directed mutagenesis by placing a stop codon at amino acid positions 272, 257 and 249 respectively, to generate Cx45-Δ272, Cx43-Δ257 and Cx40-Δ249. The primers used for generating CT truncated mutants are listed in table S1.

For creating CT-chimeric connexins, two step PCR based site directed mutagenesis was used according to previously published protocol [24]. Briefly, in the first PCR reaction, the Cx's CT-domain that has to be ligated with other Cx, was used as a c-DNA template. Primers having complementary sequences of both Cxs were used in the first PCR reaction. The amplified products of first PCR reaction bearing the complete CT part, was purified with the help of gel extraction kit (Qiagen, Germany) and used as mega-primers for the second PCR cycle. The Cx that contributes the N-terminus (NT) was used as template in the second PCR cycle to generate the CT-chimera. Constructs were confirmed by sequencing. The primer sets used for generating different chimeras are listed in table S2. Cx43-C40 represents chimeric Cx43 with the CT of Cx40; other chimeras are designated similarly.

### Cell culture and transfection

N2a cells were obtained from National Centre for Cell Science, India. For routine maintenance, cells were grown in Dulbecco's Modified Eagle's Medium (DMEM), supplemented with 10% heat inactivated foetal bovine serum and 100 units/ml antimycotic and antibiotic mixture (Gibco, USA). Cells were maintained at 37°C with 5% CO_2_ in a humidified incubator (Thermo Scientific, USA). For patch clamp recording, cells were grown on size-0 glass cover slips (Himedia Labs, India). Cells were transfected with desired Cx c-DNA constructs using Lipofectamine-2000 (Invitrogen, USA) in serum free media. After 4-5 hours of transfection, the serum free media was replaced with normal growth media containing 10% serum. All the experiments were performed after 24-36 hours of transfection.

### Oxygen glucose deprivation (OGD)

Ischemia was simulated by exposing the cells to OGD, as described in earlier reports [25]–[27]. Cells grown on cover slips, were perfused first with bicarbonate external solution (ES; pH 7.4) containing (in mM) 124 NaCl, 4 KCl, 26 NaHCO_3_, 1.5 NaH_2_PO_4_, 1.5 MgSO_4_, 10 D-Glucose, 2 CaCl_2_. The solution was continuously bubbled with 5% CO_2_ and 95% air, at room temperature (22–24°C). OGD was created by replacing the ES with ischemic solution (IS). IS has the same composition as ES with the exception of glucose being replaced with sucrose. IS was degassed for 1 hour followed by continuous bubbling with mixed gas containing 5% CO_2_ and 95% of Argon. To assure complete removal of dissolved O_2_, O_2_ scavenger sodium dithionite (2 mM) was added. Complete removal of dissolved O_2_ was confirmed by analysing the IS with a dissolved O_2_ analyser (Mettler Toledo, USA). To study the effect of ischemia, cells were exposed to IS for 5 minutes.

### Patch clamp recording

For electrophysiology, N2a cells on cover slips were transferred to a recording chamber (RC-26G, Warner Instruments, USA), mounted on the stage of an Olympus IX71 inverted microscope, attached with EMCCD camera (Andor Technology, UK). The chamber was continuously perfused with ES. For measuring junctional conductance, cell pairs were patched with two Axopatch 200B (Molecular devices, USA) amplifiers. Thin walled glass pipettes of resistances 3–5 MΩ were prepared using pipette puller P-97 (Sutter Instrument Company, USA). The pipette solution contained (in mM) 10 NaCl, 140 KCl, 1 MgCl_2_, 0.2 CaCl_2_, 3 Mg-ATP, 5 HEPES (pH 7.2), 2 EGTA, unless otherwise mentioned. After obtaining whole cell, both cells were held at 0 mV. Holding potentials of cell-1 and cell-2 are designated as V_1_ and V_2_ respectively. To create junctional voltage gradient (V_j_ = V_1_−V_2_), V_1_ was stepped to different voltages levels, keeping the V_2_ at 0 mV. The current recorded from cell-2, represents the junctional current (I_j_ = −I_2_). The current traces were low pass filtered at 1 kHz and sampled at 10 kHz with the help of Digidata 1440 (Molecular devices, USA).

In OGD experiments, V_j_ of±15 mV was applied to cell-1 for 5 second, with 5 second recovery step at 0 mV in between the pulses. The I_j_, obtained from cell-2, was normalized to 15 mV of V_j_, at every 10^th^ second to calculate G_j_. The maximum conductance, G_jmax_, of each experiment was used to normalize G_j_. The mean±SEM of normalized G_j_ generated from 5–7 independent experiments were plotted against time, to check the degree of uncoupling due to OGD treatment.

To determine the voltage sensitivity of different Cxs, step protocol with an increment of 20 mV was applied to cell-1 ranging from −120 mV to +120 mV for 20 s. There was a pause of 30 s at 0 mV between two sweeps. I_j_s obtained in response to 10 mV pre pulse were used to normalize the respective current traces. The normalized steady state G_j_ (G_j(ss)_) was plotted with respect to V_j_. To calculate voltage dependency, G_j(ss)_−V_j_ plot was fitted with two-states Boltzmann's equation that assumes channel gating is a two steps process. The following form of Boltzmann's equation was used: G_j(ss)_ = [(G_jmax_−G_jmin_)/{1+exp[A(V_j_−V_0_)]}]+G_jmin_, where G_jmax_ is the normalized maximum conductance (equal to 1), G_jmin_ is the minimum conductance obtained at higher V_j_s, V_0_ is the voltage at which G_j(ss)_ is half maximal, A is the slope factor of the curve that can be defined as *zq/kT*, where z is valance of charge q that acts as voltage sensor for the transition from open to closed state. k and T represent Boltzmann's constant and absolute temperature, respectively.

Relaxation kinetics of WT and CT truncated mutants were compared to check the effect of truncation on channel gating. I_j_ traces (recorded at −100 mV, V_j_) were fitted with standard single or double exponential equation by using pCLAMP 10.

### Calcium imaging

Intracellular calcium was measured ratio-metrically with Fura-2, as described before [28]. Briefly, adherent N2a cells on cover slips were incubated with 10 µM Fura-2-AM (Molecular Probes, USA) for 30 minutes at room temperature (22–24°C), in solution containing (in mM) 140 NaCl, 5 KCl, 1 MgCl_2_, 10 HEPES (pH 7.4), 10 D-glucose, 2 CaCl_2_. After 30 minutes of incubation, cells were washed in fura-2 free buffer for 30 minutes. Fura-2 loaded cells were illuminated with dual excitation wave lengths of 340 nm and 380 nm, while capturing the emission signal at 510 nm with the help of appropriate excitation and emission filters (Chroma Technology, USA). Fast excitation switching was done by Lambda DG4 wave length switching system (Sutter Instrument Company, USA). Images were acquired with an EMCCCD camera (Andor Technology, UK) attached with Olympus IX71 microscope, controlled through IQ software. Images were acquired at every 5 second interval for about 10 minutes duration. There was no detectable bleaching of the dye during entire time course of the experiment. F340/F380 ratio was calculated from background subtracted images. Inclusion of 2 mM BAPTA in pipette solution interfered with fura-2AM based calcium imaging. In presence of BAPTA, Fura-2 fluorescence signal became erratic. To avoid this, Rhod-2 was used to detect [Ca^2+^]_i_ when BAPTA was present in pipette solution. Rhod-2AM loading process was similar to Fura-2-AM loading.

### Intracellular pH (pH_i_) measurement

pH_i_ was measured with 2',7'-Bis-(2-Carboxyethyl)-5-(and-6)-Carboxyfluorescein dye (BCECF) as described earlier [29], [30]. Briefly, cells were incubated with 10 µM of BCECF-AM (Molecular Probes, USA) for 10 minutes in the same buffer, used for fura-2-AM loading, at room temperature (22–24°C). Experiments were performed after 10 minutes of washing in BCECF free buffer. BCECF loaded cells were excited at 440 nm and 490 nm; emission was captured at 530 nm using appropriate filter set (Chroma technology, USA). The ratio of the emitted intensity (F490/F440) was calculated off line after subtracting the back ground. Calibration of the ratio values (F490/F440) into pH_i_ was done by the equation:

pH = log {[(R/R_pH 7_)−R_min_]/[R_max_−(R/R_pH 7_)]}+pK

Where R is the ratio (F490/F440), R_pH 7_ is the ratio at pH 7, R_min_, R_max_ and pK are the minimum ratio, maximum ratio and negative log of the dissociation constant respectively. For calibration, BCECF loaded cells were perfused with high K^+^ solution of different pH ranging from 4 to 9, containing (in mM) 105 Potassium Aspirate, 4 KCl, 1.5 KH_2_PO_4_, 1.5 MgSO_4_, 49 NMDG, 10 EGTA, 10 glucose, 10 HEPES with 0.01 nigericin (Invitrogen, USA). The average (n = 35) ratio values (490 nm/440 nm) obtained from pH 4 to pH 9 were fitted with non-linear logistic equation to determine the values of R_min_, R_max_ and pK. The value of R_pH = 7_ was obtained at the end of each experiment, by adding 10 µM nigericin to high K^+^ solution of pH 7.

For clamping pH_i_ at a particular value during OGD, pipette solution with increasing concentrations of HEPES (5, 10, 20, 50 & 80 mM) was used. With increasing concentration of HEPES, equimolecular amount of KCl was removed to maintain the osmolarity. The pipette solutions were usually supplemented with 50 µM BCECF free acids (Molecular Probes, USA) to avoid dilution of the BCECF dye inside the cell. At the onset of the experiments, the pipette solutions were adjusted to pH 7.2 with KOH.

### Immunofluorescence

Cells were fixed with 4% paraformaldehyde for 20 minutes, washed thoroughly with 1X phosphate buffer saline (PBS), (GIBCO, USA), followed by blocking with 10% FBS and 0.3% triton X-100 in 1X PBS, for 1 hour. After blocking, cells were incubated overnight with primary antibody (1∶100 dilutions) at 4°C. Cells were counter stained with secondary antibody conjugated with FITC or Alexa-594 (1∶500) for one hour. Nucleus was stained with 1 µM DAPI (Molecular Probes, USA). Rabbit-anti Cx45 (N-terminal) primary antibody (LS-C14577-life span bio sciences, USA) and anti-rabbit-594 secondary antibody (A11080-Invitrogen, USA) were used for Cx45, Cx45-Δ272, Cx45-C43 and Cx45-C40 transfected N2a cells. Mouse-anti Cx43 (D-7) primary antibody (Sc-13558-Santa Cruz Biotechnology, USA) and anti-mouse-FITC secondary antibody (Sc-2010-Santa Cruz Biotechnology, USA) were used for Cx43, Cx43-Δ257, Cx43-C40 and Cx43-C45 transfected cells. Rabbit anti-Cx40 primary antibody (36-5000-Invitrogen, USA) and anti-rabbit-FITC secondary antibody (Sc-2012-Santa Cruz Biotechnology, USA) were used for Cx40, Cx40-Δ249, Cx40-C43 and Cx40-C45 transfected N2a cells.

Zeiss laser scanning confocal microscope (Carl Zeiss, USA) was used to acquire the images. Minimum of 20–25 pair transfected cells having junctional plaques were captured. The diameter of the junctional plaques, were measured with the help of ZEN light edition 2009 software (Carl Zeiss, USA).

### Statistical analysis

Data provided here are representative of 3–5 independent experiments. Values are mean±SEM of 5–11 replicates as described in the respective figure legends. Student paired t-test and one way ANOVA were performed for comparison between two and multiple groups respectively.

## Results

### Biophysical characterization of wild type and eGFP tagged gap junctions

**Figure 1:**
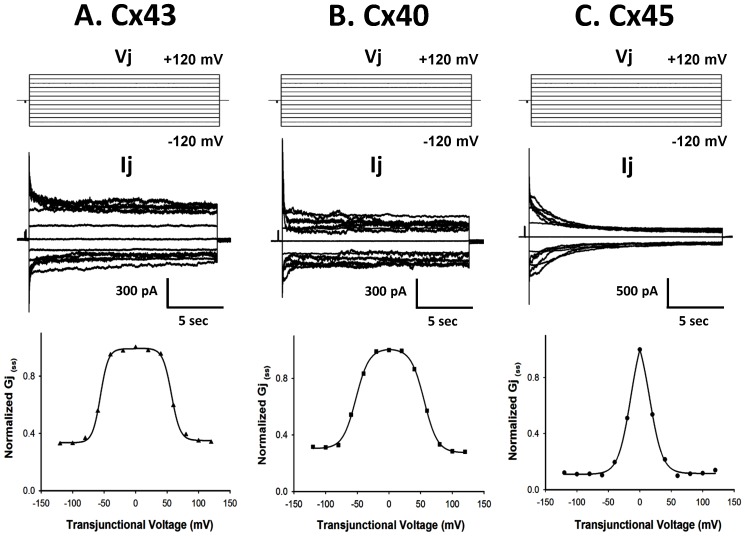
Sensitivity of Cx43, Cx40 and Cx45 to junctional Voltage (V_j_). Upper panel shows the voltage step protocol. V_j_ steps of 20 mV increment, from −120 to +120 mV were applied for 20 sec followed by 30 sec recovery at 0 mV. A short pre pulse (100 msec) of 10 mV was applied at the beginning of each pulse to normalize G_j_. Middle panel: representative I_j_ traces. Lower panel: normalized G_j(ss)_ versus V_j_ plot. Two states Boltzmann fitted curve, represented by the solid line was generated with pCLAMP 10 software.

Wild type Cx43, Cx40 and Cx45 readily formed GJs when expressed in N2a cells. Figure 1 shows the representative current traces at different junctional voltages (V_j_), recorded from the cell pair containing homotypic junctions of Cx43, Cx40 and Cx45. The average junctional conductance (G_j_) for Cx43, Cx40 and Cx45 were 60±4 nS (n = 45), 43±5 nS (n = 46) and 36±4 nS (n = 48) respectively (Table 1). G_j(ss)_−V_j_ dependence of Cx43, Cx40 and Cx45 are presented in the lower panel of figure 1. A 10 mV pre-pulse was applied before every voltage step to normalize the G_j(ss)_. The normalized G_j(ss)_ (with respect to 10 mV pre pulse) versus V_j_ plots were fitted with two state Boltzmann equation to determine the voltage sensitivity. The Boltzmann parameters are presented in table 2. V_0_ represents the V_j_ at which G_j(ss)_ is reduced by 50%. In agreement with previous reports, V_0_ for Cx45 (+11±1.8 and −11±1.5) was lowest among all three Cxs (Cx43: +57±0.5 and −56±0.5, Cx40: +50±0.7 and −49±1.2), indicating its highest V_j_ sensitivity. The V_j_ sensitivity followed the order Cx45>Cx40>Cx43.

**Table 1:** Average conductance and junctional plaque diameter of different Connexins.

Connexin	Average Conductance, nS (n)	Average plaque diameter, µM (n)
Cx45 (WT)	36±3.5 (48)	3.8±0.3 (30)
Cx45-Δ272	3±0.5 (23)	ND (20)
Cx45-C43	49±4.6 (37)	5.8±0.3 (35)
Cx45-C40	37±4.3 (33)	2.3±0.1 (33)
Cx43 (WT)	60±4.4 (45)	7.0±0.4 (32)9.6±0.5 (35)
Cx43-Δ257	72±4.6 (39)	
Cx43-C40	59±3.2 (33)	6.6±0.4 (30)
Cx43-C45	NF	NF
Cx40 (WT)	43±4.6 (46)	4.9±0.3 (27)
Cx40-Δ249	4±0.5 (20)	ND (15)
Cx40-C43	40±3.6 (35)	3.4±0.2 (30)
Cx40-C45	NF	NF

WT: wild type; ND: not detectable; NF: not functional; data presented are mean±SEM.

**Table 2:** Boltzmann fitting parameters for different Connexins.

Connexin	G_min_±V_j_ (mV)	V_0_±V_j_ (mV)	A	τ_1_ and τ_2_, (ms)
Cx45 (WT)	0.10±0.02	+11±1.8	11.3±0.9	37±4
(n = 7)	0.11±0.01	−11±1.5	12.4±0.7	1677±111
Cx45-eGFPN1	0.10±0.01	+12±1.7	11.9±0.9	40±6
(n = 7)	0.10±0.02	−11±2.1	12.7±1.0	1593±125
Cx45-Δ272	0.12±0.01	+12±2.2	10.2±1.9	31±3
(n = 5)	0.12±0.02	−12±2.1	10.4±1.3	1571±110
Cx45-C43	0.12±0.01	+11±2.3	14.8±0.9	50±3
(n = 7)	0.12±0.01	−12±1.7	15.7±1.7	1691±50
Cx45-C40	0.22±0.01	+18±1.9	9.1±1.5	144±11
(n = 7)	0.23±0.02	−18±1.7	9.4±1.3	1895±119
Cx43 (WT)	0.35±0.01	+57±0.5	7.9±0.7	70±5
(n = 7)	0.33±0.01	−56±0.5	8.4±1.2	931±54
Cx43-eGFP	0.25±0.02	+62±1.4	11.5±1.4	...
(n = 5)	0.28±0.01	−64±1.2	11.1±1.1	1804±193
Cx43-Δ257	0.29±0.01	+59±0.9	8.7±0.8	...
(n = 3)	0.27±0.01	−58±0.7	8.9±1.1	2234±176
Cx43-C40	0.31±0.01	+62±0.7	11.3±0.4	99±7
(n = 5)	0.32±0.01	−61±0.6	10.6±0.6	1046±68
Cx40 (WT)	0.26±0.02	+50±0.7	13.1±0.7	86±12
(n = 6)	0.27±0.01	−49±1.2	12.5±1.0	824±66
Cx40-Δ249	0.20±0.01	+51±0.8	14.1±0.7	91±15
(n = 3)	0.19±0.01	−51±0.5	12.2±0.5	...
Cx40-C43	0.26±0.02	+51±2.0	14.4±1.9	107±5
(n = 7)	0.29±0.01	−50±1.8	12.6±1.6	1078±69

G_min_: minimum conductance; V_j_: junctional voltage; A: slope factor. For calculating time constants ι_1_ and ι_2_ of voltage desensitization, the junctional current decay in response to 100 mV voltage step, was fitted with mono or double exponential function.

GJs are often studied by attaching different fluorescent proteins at the CT of Cx [31]. We analysed the V_j_ sensitivity of Cx43-eGFP and Cx45-eGFP as these were used in our study. As shown in figure S1, G_j(ss)_−V_j_ plot of Cx45-eGFP is indistinguishable from the wild type Cx45. On the other hand, G_j(ss)_−V_j_ plot slightly shifted towards higher voltage upon attachment of eGFP with Cx43 indicating the decrease of voltage sensitivity, which is consistent with earlier reports [31]. V_0_ (in mV) for Cx43 were +57±0.5 and −56±0.5, whereas for Cx43-eGFP the values were +62±1.4 and −62±1.2 respectively (Table 2). Cx40-eGFP was non-functional for reasons unknown and thus not used in our study.

### Ischemia uncoupled Cx43, Cx40 and Cx45 to different extents

**Figure 2:**
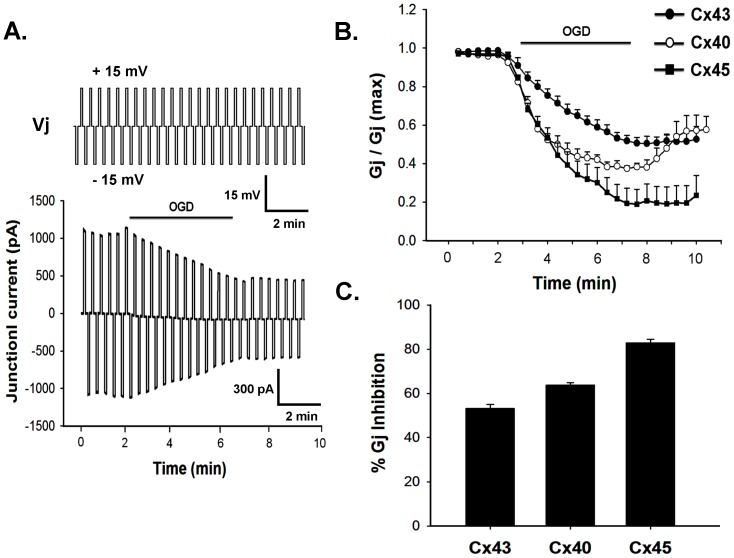
Effect of OGD on different cardiac gap junctions. **A**. representative I_j_ trace of Cx43 gap junction recorded from transfected N2a cells. I_j_ decreased at the onset of OGD. Solid line above the current trace represents duration of OGD. Voltage protocol is presented on top of the current trace. **B**. OGD reduced the G_j_ (normalized) of all gap junctions. Cx45 showed maximum reduction. Values are the mean±SEM. **C**. quantitative representation of the data obtained from panel B.

**Figure 3:**
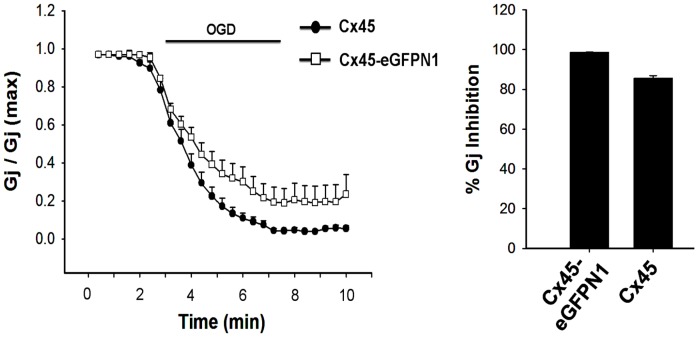
OGD inhibited Cx45-eGFP more than Cx45. OGD uncoupled eGFP tagged Cx45 almost completely, compared to 85±2% inhibition in wild type Cx45. The difference is statistically significant (p<0.001).

Ischemia was simulated by exposing the cells to OGD for 5 minutes. Cell pairs expressing Cx43, Cx40 or Cx45 were voltage clamped with two patch pipettes in whole cell configuration. Junctional current (I_j_) from cell-2 was monitored continuously in external solution (ES), bubbled with 5% CO_2_+95% air, by stepping cell-1 from holding potential, 0 mV to±15 mV (for 5 sec) in every 5 sec interval. Cell-2 was clamped at 0 mV. After confirming that there was no spontaneous run down of I_j_, ES was replaced with ischemic solution (IS), bubbled with 5% CO_2_+95% argon. After 5 minutes, IS was replaced back with ES. When cells were exposed to OGD, I_j_ of all Cxs reduced immediately. I_j_ reached a steady value within 5 minutes and did not decrease further under prolonged OGD treatment. Figure 2A shows the representative I_j_ of Cx43, which decreased to almost 50% within 5 minutes of OGD. Interestingly the extent of uncoupling varied among Cxs. As shown in figure 2B and 2C, Cx43 was quite resistant to OGD in comparison to Cx45 and Cx40. Cx45 showed the highest sensitivity to OGD. In case of Cx45, G_j_ decreased by 85±2% (n = 9) in 5 minutes OGD, whereas there was only about 53±3% (n = 7) reduction of G_j_ for Cx43 and 64±1% (n = 7) for Cx40. The rate of uncoupling also varied among Cxs. As shown in figure 2B, Cx43 uncoupled at a much slower rate in comparison to Cx45 and Cx40. Tagging of GFP to CT of some Cxs has been reported to alter their properties. For example, Cx45-eGFP, failed to rescue the Cx45 knockout mice from embryonic lethality [32]. We compared the susceptibility of Cx43-eGFP and Cx45-eGFP to OGD with their wild type counterpart. Effect of OGD on Cx43-eGFP was same as Cx43 (data not shown). However Cx45-eGFP showed higher sensitivity to OGD. As shown in figure 3, G_j_ of Cx45-eGFP reduced by 99±0.2% (n = 8) in 5 minutes of OGD treatment.

### [Ca^2+^]_i_ rises and pH_i_ decreases during simulated ischemia

**Figure 4:**
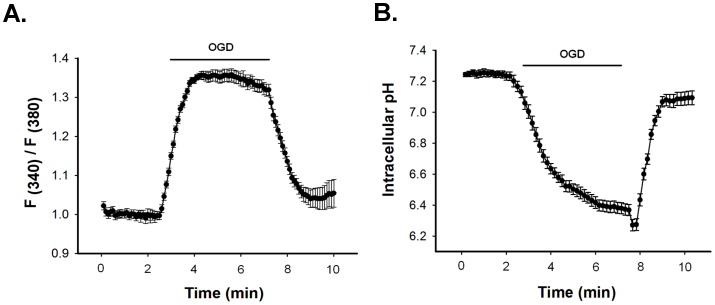
OGD increases intracellular calcium ([Ca^2+^]_i_) and decreases intracellular pH (pH_i_). OGD induced rise of [Ca^2+^]_i_ and decrease of pH_i_ in N2a cells are shown in A and B. [Ca^2+^]_i_ and pH_i_ were measured ratio-metrically using Fura-2 and BCECF dye. Fluorescence intensity was calculated from background subtracted images using ANDOR IQ programme. Values are the mean±SEM of 20–25 cells.

Intracellular acidification and [Ca^2+^]_i_ rise are known to be associated with ischemia [26]. Fura-2 loaded N2a cells when exposed to OGD, [Ca^2+^]_i_ increased immediately and maintained a steady value through the entire duration of OGD (Figure 4A). [Ca^2+^]_i_ returned to basal level when IS was replaced with ES. F340/F380 increased to 1.6±0.2 from 0.92±0.1 (n = 25). Cx transfected cells also showed [Ca^2+^]_i_ rise to the same extent and there was no difference among Cxs (data not shown).

pH_i_ was estimated ratio-metrically with BCECF. pH_i_ dropped from 7.25±0.1 to 6.35±0.4 (n = 20) in 5 minutes of OGD. pH_i_ was restored once IS was replaced with ES (Figure 4B). The degree of intracellular acidification does not depend on the level Cx expression. The pH_i_ of untransfected cells also dropped to the same extent following 5 minutes of OGD (data not shown).

### Role of [Ca^2+^]_i_ and pH_i_ in uncoupling

**Figure 5:**
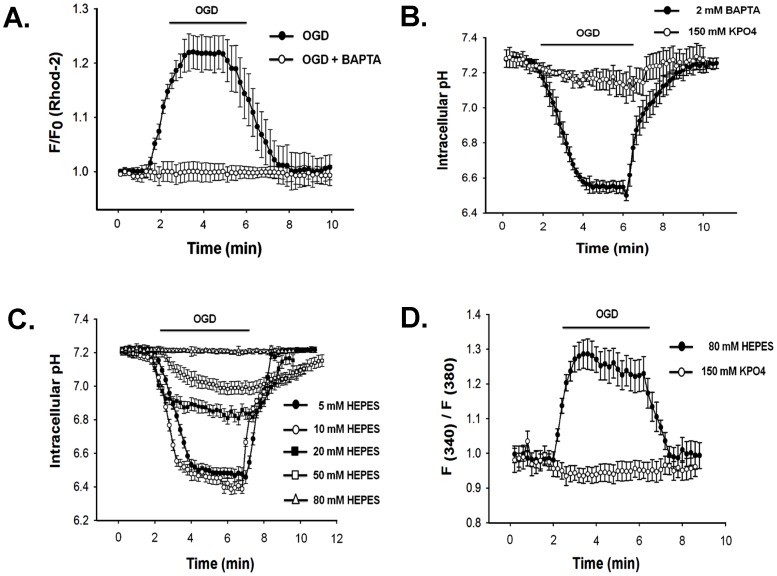
Clamping of intracellular calcium ([Ca^2+^]_i_) and (pH_i_) during OGD. **A**. Addition of 2 mM BAPTA in the pipette solution restricted [Ca^2+^]_i_ rise during OGD. Rhod-2 dye was used to determine [Ca^2+^]_i_ in presence of BAPTA. **B**. 2 mM BAPTA did not affect OGD induced acidosis, whereas 150 mM KPO_4_ in the pipette, prevented pH change. **C**. OGD induced change of pH_i_ in presence of different concentration of HEPES in the pipette. 80 mM HEPES prevented acidosis completely. pH_i_ was determined ratiometrically using BCECF. **D**. 80 mM HEPES in the pipette, did not affect OGD-induced [Ca^2+^]_i_ rise. 150 mM KPO_4_ prevented both calcium rise and acidosis (B). [Ca^2+^]_i_ was measured with fura-2. Values are the mean±SEM recorded from 5–10 cells.

**Figure 6:**
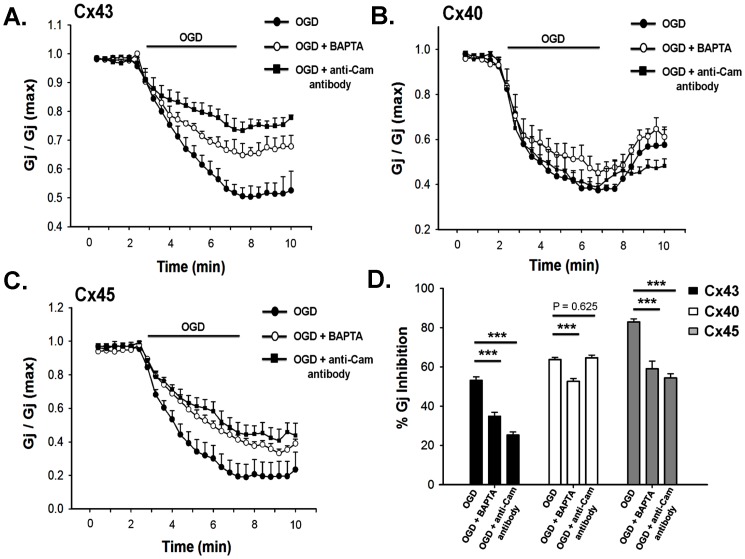
Role of intracellular calcium and calmodulin (CaM) on OGD induced uncoupling of gap junctions. **A**. OGD induced uncoupling of Cx43 was prevented partially by intracellular administration of BAPTA (2 mM in pipette) and anti-CaM antibody (7 µM in pipette). Anti-CaM antibody showed greater protection than BAPTA. **B**. anti-CaM antibody had no effect on the uncoupling of Cx40. BAPTA attenuated the reduction of G_j_ significantly, but to a lesser extent. **C**. Both BAPTA and anti-CaM antibody reduced the uncoupling of Cx45 to the same extent. **D**. quantitative representation of the data obtained from A, B and C. ***, p<0.001.

The contributions of [Ca^2+^]_i_ and pH_i_ in ischemic uncoupling of GJ were evaluated individually as well as in combination, by restricting their change during OGD. To clamp [Ca^2+^]_i_, 2 mM BAPTA was included in the pipette solution. After obtaining ‘whole cell’, cells were allowed to equilibrate with BAPTA for 5 minutes. Intracellular administration of BAPTA prevented OGD-induced [Ca^2+^]_i_ rise (Figure 5A) and attenuated the reduction of G_j_ of all Cxs significantly (Figure 6). After 5 minutes of OGD, G_j_ of BAPTA treated cell pair reduced by: 35±2% (n = 6) for Cx43, 52±2% (n = 6) for Cx40 and 59±4% (n = 7) for Cx45. In control experiment (without BAPTA), G_j_ of Cx43, Cx40 and Cx45 decreased by 53±3%, 64±1% and 85±2% respectively. It is evident that [Ca^2+^]_i_ has lesser contribution in the uncoupling of Cx40 in comparison to Cx43 and Cx45. Effect of BAPTA is independent of pH_i_ changes. It did not affect intracellular acidification during OGD (Figure 5B).

**Figure 7:**
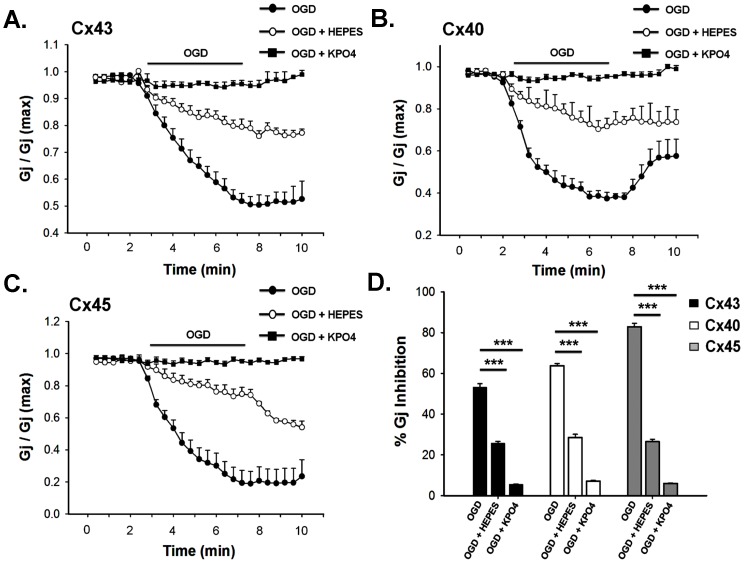
Restriction of intracellular acidification and calcium rise prevents OGD-induced uncoupling of gap junctions. Intracellular pH (pH_i_) was maintained to 7.2 during OGD by adding 80 mM HEPES in the pipette solution. Clamping of pH_i_ alone attenuated the reduction of Gj of all Cxs i.e. Cx43, Cx40 and Cx45 significantly **(A, B and C)**. But restriction of both [Ca^2+^]_i_ elevation and acidification by adding 150 mM potassium phosphate in the pipette solution prevented the uncoupling of all gap junctions completely **(A, B and C)**. **D**. statistical representation of data generated from 6–9 independent experiments. Values are the mean±SEM. ***, p<0.001.

To study the role of acidification on the uncoupling of GJ, we restricted the pH_i_ change by using high concentration of HEPES in pipette solution. As shown in figure 5C, gradual increase of HEPES concentrations from 5 mM to 80 mM, prevented the pH_i_-drop in a graded fashion. 80 mM of HEPES completely prevented acidification and pH_i_ was maintained at 7.2 throughout the OGD. However it did not prevent the [Ca^2+^]_i_ rise, as represented in figure 5D. Like clamping of [Ca^2+^]_i_, when pH_i_ was maintained to 7.2, all Cxs showed lesser vulnerability to ischemic uncoupling. Reduction of G_j_ was prevented significantly (Figure 7). pH_i_-clamped cell pair showed the reduction of G_j_: 26±1% (n = 7) for Cx43, 29±2% (n = 6) for Cx40 and 27±1% (n = 7) for Cx45.

To evaluate the cumulative role of [Ca^2+^]_i_ and pH_i_, we clamped both by injecting high concentration of potassium phosphate buffer. Addition of 150 mM KPO_4_ in pipette solution prevented acidification as well as [Ca^2+^]_i_ rise during OGD, as shown in figure 5B and 5D. When [Ca^2+^]_i_ and pH_i_ were clamped, OGD could not uncouple any of the junctions as observed by the steady G_j_ throughout (Figure 7). Cx43, Cx40 and Cx45 became completely resistant to OGD.

### Calmodulin (CaM) is involved in ischemic uncoupling

CaM has been reported to modulate both voltage and chemical gating of gap junctions [33], [34]. CO_2_ mediated uncoupling of Cx45 was prevented by suppressing the expression CaM with antisense RNA [35]. We studied the role of CaM in ischemic uncoupling. Anti-CaM antibody (Sigma-Aldrich, USA) was injected into the cell pair through patch pipette by supplementing pipette solution with 7 µM of antibody. Antibody was allowed to diffuse for 5 minutes after obtaining whole cell and I_j_ was recorded. As shown in figure 6, the antibody attenuated ischemic uncoupling of Cx43 and Cx45, GJs significantly. However, uncoupling of Cx40 by OGD was not affected at all by anti-CaM antibody. G_j_ of the antibody treated Cx43 expressing cell pair reduced by 26±2% (n = 7) in response to 5 minutes OGD, whereas in control (without anti-CaM antibody) the reduction was 53±3%. In case of Cx45, antibody treated and the control cell pair showed the reduction of G_j_ by 54±2% (n = 8), and 85±2% respectively (Figure 6D).

### Role of carboxy terminal (CT) tail in ischemic uncoupling

#### Properties of CT deletion mutants

CT of Cxs has been implicated in many physiological and pathophysiological processes [22], [32], [36]. To study the role of CT in ischemic uncoupling, we generated CT-deletion mutants Cx43-*Δ*257, Cx40-*Δ*249 and Cx45-*Δ*272 (see material and methods). Deletion of CT impaired the G_j_ of Cx40 and Cx45. The average G_j_ of Cx40 reduced from 43±4.6 nS (n = 46) to 4±0.5 nS (n = 20) upon deletion of CT. Similarly, Cx45-*Δ*272 showed average G_j_ of 3±0.5 nS (n = 23) which is about 12 fold lesser than wild type (36±3.5 nS, n = 48), (Table 1). Consistence with earlier reports, Cx43-*Δ*257 displayed higher average G_j_ and larger plaque diameter than the wild type (Table 1). Deletion of CT also altered the gating parameters of GJs. In accordance with a previous report, removal of CT from Cx43 and Cx40 did not alter their sensitivity to V_j_ (Table 2) as V_0_ remained unaltered. However there was a small change of minimum conductance (G_min_) and the desensitization kinetics altered significantly (Table 2). To study the desensitization kinetics, V_j_ was stepped to -100 mV. The decay of I_j_ was fitted with either single or double exponential function as required. The time constants for relaxation, τ_1_ and τ_2_, are presented in figure S2 and table 2. Most GJs are known to follow two gating processes: slow gating and fast gating [31], [37], [38]. In accordance with previous reports [37], [38], all wild type GJs in our experiment showed double exponential decay of I_j_ (Figure 2 and Table 2). Fast time constant and slow time constant of voltage relaxation reflect closure of fast gate and slow gate respectively [37]. Cx43-*Δ*257 and Cx40-*Δ*249 followed monophasic decay compared to biphasic relaxation of the corresponding wild types, suggesting loss of one gate in both cases [37], [38]. Interestingly removal of CT did not affect the gating of Cx45 as both slow and fast τ remained unaltered (Figure S2 and Table 2).

**Figure 8:**
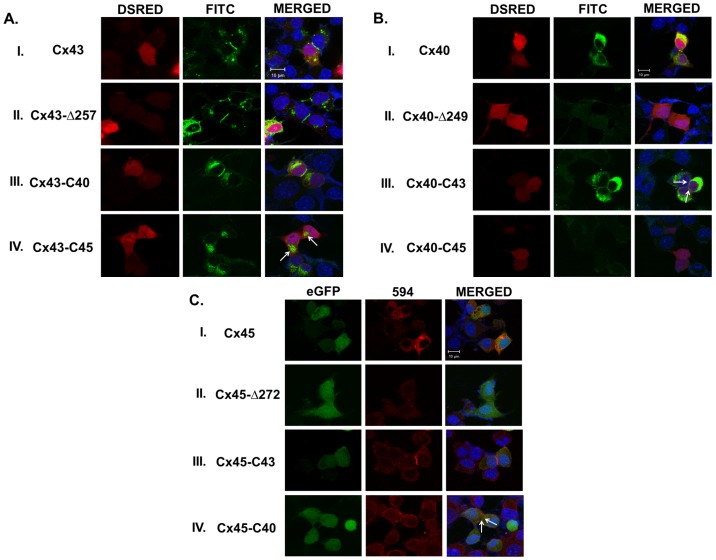
Carboxy terminal (CT) domain of connexins, regulate junctional plaque formation. **A**. I, II, III and IV are the representative confocal microscopic images of N2a cells expressing Cx43, Cx43-Δ257, Cx43-C40 and Cx43-C45 respectively. Junctional plaques formed by Cx43-Δ257 were significantly bigger (p<0.05) than that of wild type Cx43. There was no significant difference in plaque diameter between Cx43-C40 and Cx43. Cx43-C45 did not form detectable plaque. Most of the connexins were detected in the perinuclear area (shown with white arrows). **B**. I, II, III and IV are the images of Cx40, Cx40-Δ249, Cx40-C43 and Cx40-C45 expressing cells respectively. Cx40-Δ249 plaques were not detectable. Plaques formed by Cx40-C43 were thin and punctuated (shown with white arrows). Cx40-C45 did not form detectable plaque. **C**. I, II, III and IV, images of Cx45, Cx45-Δ272, Cx45-C43 and Cx45-C40 transfected N2a cells respectively. Cx45-Δ272 junctional plaques were not detectable. Cx45-C43 plaques were significantly larger than corresponding Cx45 plaques. Cx45-C40 plaques were punctuated and smaller compared to wild type Cx45.

Immunofluorescence analysis revealed the characteristics of junctional plaque formed by different CT-truncated mutants (Figure 8). Both wild type and Cx43-*Δ*257 showed bright fluorescent plaque at the cell-cell junction. Average diameter of the Cx43-*Δ*257 junctional plaque is significantly bigger than the wild type Cx43 (Table 1), which is in agreement with the observed higher G_j_ of Cx43-*Δ*257. Although clear junctional plaques were detected in cell pairs containing wild type Cx40 or Cx45, it was not visible in corresponding CT truncated mutants (Figure 8); a possible reason for their very low G_j_.

### Effect of OGD on CT truncated mutants

**Figure 9:**
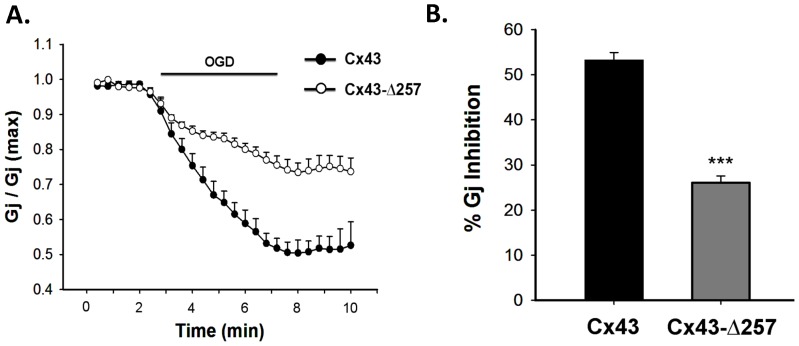
OGD induced uncoupling of CT truncated Cx43 (Cx43-**Δ** **Δ**257). **A**. G_j_ of CT truncated Cx43 reduced to a lesser extent compared to wild type Cx43, in response to OGD. **B**. quantitative representation of data generated from 5–7 independent experiments. Values are the mean±SEM. ***, p<0.001.

Cx43-*Δ*257 when exposed to OGD, showed significant resistance to uncoupling. 5 minutes of OGD resulted in only 26±2% (n = 5) reduction of G_j_, compared to 53±3% in wild type (Figure 9), suggesting the involvement of Cx43-CT in ischemic uncoupling. OGD on deletion mutants of Cx40 and Cx45 could not be performed due to their low average G_j_.

We observed that among 3 cardiac Cxs, Cx45 is the most sensitive to OGD, whereas Cx43 is the least one. To check if this variability is conferred by the CT, we generated several Cx chimeras e.g. Cx43-C45, Cx43-C40, Cx40-C45, Cx40-C43, Cx45-C43 and Cx45-C40 by swapping CT. Biophysical properties of different chimeras and their sensitivity to OGD are described below.

### Biophysical properties of CT chimeras

Deletion of the CT of Cx40 and Cx45 attenuated their G_j_ by more than 10 fold (Table 1). CT truncated mutants Cx40-*Δ*249 and Cx45-*Δ*272 did not assemble properly to form junctional plaque as evident from confocal microscopy. When CT of Cx43 was tethered to truncated Cx40 and Cx45, it rescued their G_j_ completely (40±3.6 nS, n = 35 for Cx40-C43 and 49±4.6 nS, n = 37 for Cx45-C43), (Table 1). Cx45-C43 even showed higher G_j_ than wild type Cx45 (36±3.5, n = 48). Also, both the chimeras showed bright junctional plaques with diameters comparable to their wild type counterpart (Figure 8 and Table 1). Unlike other Cxs, truncation of CT significantly improved the G_j_ of Cx43 (60±4.4 nS, n = 45 for Cx43 and 72±4.6 nS, n = 39 for Cx43-*Δ*257, p<0.05), (Table 1). Tethering of CT of Cx40 to the Cx43-*Δ*257 restored its G_j_ to the wild type value (Table 1). CT of Cx40 also rescued Cx45-*Δ*272. Cx45-C40 chimera behaved like wild type Cx45 in terms of G_j_ (37±4.3 nS, n = 33 for Cx45-C40 and 36±3.5 nS, n = 48 for Cx45), though the plaque diameter was slightly smaller (Figure 8 and Table 1). Interestingly, CT of Cx45 was not compatible with either Cx43 or Cx40. None of the chimeras with CT of Cx45 formed functional channels (Figure 8 and Table 1).

The voltage sensitivity and relaxation kinetics of different CT chimeras was studied by step protocol. The results are shown in table 2. Except Cx45-C40, voltage sensitivity of all chimeras resembled their respective wild type. V_0_ of Cx45-C40 shifted towards higher voltage indicating decrease of V_j_ sensitivity. Like wild type Cx, voltage relaxation of all chimeras showed biphasic decay of I_j_. In comparison to wild type, all chimeras except Cx45-C40 showed slower decay as indicated by higher τ_1_ and τ_2_. τ_1_and τ_2_ of Cx45-C43 are not significantly different from wild type Cx45.

### Ischemic uncoupling of CT chimeras

**Figure 10:**
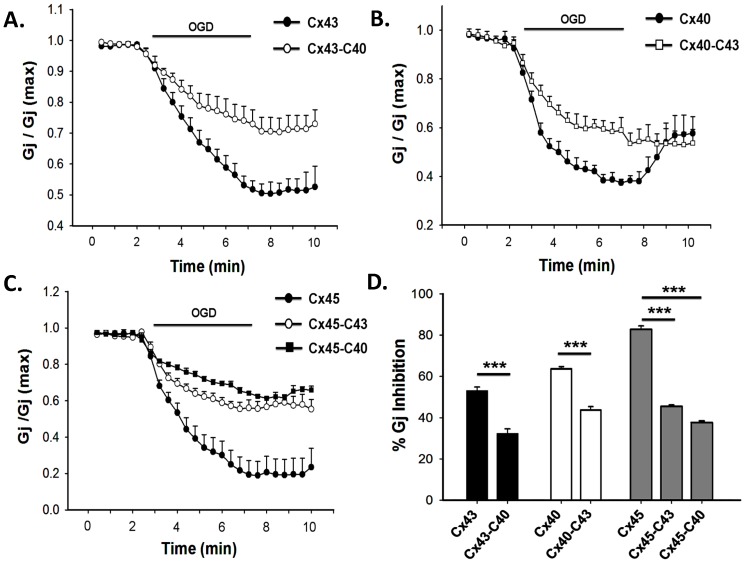
Effect of OGD on gap junctions made of different chimeric connexions. **A**, chimeric Cx43 with the CT of Cx40 (Cx43-C40) and **B**, Cx40 with the CT of Cx43 (Cx40-C43) uncoupled to a lesser extent compared to the corresponding wild type. **C**. Comparison of the uncoupling of wild type Cx45 and Cx45 chimeras containing CT of Cx40 and Cx43. **D**, statistical representation of the extent of uncoupling of different chimeras. Values are the mean±SEM of 5-7 independent experiments. ***, p<0.001.

Effect of 5 minutes OGD on different chimeras, are presented in figure 10. If the CT of a particular Cx is the sensor for ischemia, chimera would respond like the Cx that contributed the CT. Cx43-*Δ*257 showed 26±2% inhibition of G_j_ in response to OGD. Tethering of CT of Cx40 did not improve the sensitivity to OGD significantly. G_j_ of Cx43-C40 inhibited by 33±2% (n = 7), whereas wild type Cx40 exhibited 64±1% inhibitions. Similarly, CT of Cx40 though rescued G_j_ of truncated Cx45; the chimera exhibited far lesser sensitivity to OGD than Cx40 and wild type Cx45. G_j_ of Cx45-C40 reduced by 38±1% (n = 7) as shown in figure 10. Interestingly, CT of Cx43 not only rescued the G_j_ of truncated Cx45 and Cx40, it brought their OGD sensitivity closer to wild type Cx43. After 5 minutes of OGD, Cx43, Cx40-C43 and Cx45-C43 showed reduction of G_j_ by 53±3% (n = 7), 44±2% (n = 8) and 45±1% (n = 6) respectively. It suggests that unlike CT of Cx40, CT of Cx43 is partially associated with OGD mediated uncoupling.

## Discussion

Ischemia reduces gap junctional communication in many cell types. Intracellular acidification and [Ca^2+^]_i_ rise, generally observed during ischemia, are known to promote uncoupling of GJs. However the molecular mechanisms are not clearly understood. In the present study, we simulated ischemia by depriving oxygen and glucose and studied its effect on cardiac GJ channels, formed of Cx43, Cx40 and Cx45. Contribution of [Ca^2+^]_i_ and pH_i_ in uncoupling was studied. Additionally we demonstrated the role of CT of Cxs in OGD mediated uncoupling.

We studied the effect of ischemia on different cardiac gap junctions, over-expressed in N2a cells. N2a cells have been used extensively in many earlier reports for studying different connexins as they do not have endogenous gap junctions [39], [40]. Also, N2a cells are good model system for studying ischemia [41], [42]. Cardiomyocytes or cell line of cardiac origin would have been an appropriate model for studying cardiac connexins. However, cardiomyocytes express all three connexins (Cx45, Cx43 and Cx40) which may form homomeric and heteromeric channels in different combinations, thereby making it difficult to study the effect of ischemia on individual connexins. Moreover, available gap junction blockers are not very specific. Similarly, all available cell lines of cardiac origin express multiple connexins.

GJs made of Cx45, Cx43 and Cx40 varied remarkably in their biophysical properties as well as sensitivity to OGD. In accordance with earlier report [43], we showed V_j_ sensitivity follows the order Cx45>Cx40>Cx43. V_j_ sensitivity is an inherent property of GJs and determined by several factors including number of charge residues in the voltage sensor. Voltage sensors in cardiac GJs are ill defined and the underlying mechanism of differential voltage sensitivity is unknown. Voltage sensitivity of Cxs may play an important role during ischemic uncoupling. Ischemia affected cardiomyocytes, depolarize quickly which may cause development of higher V_j_ between ischemic cell and healthy cell. GJs that are less sensitive to V_j_, will remain open longer and may prevent development of arrhythmia.

OGD reduced gap junctional communication of all cardiac Cxs. However, the degree of uncoupling varied among Cxs. None of the GJs closed completely upon exposure to OGD for 5 minutes. Cx45 showed maximum reduction of G_j_, while Cx43 was most resistant to OGD. Once cells were exposed to IS, there was elevation of [Ca^2+^]_i_ and pH_i_ dropped. When [Ca^2+^]_i_ rise was prevented with BAPTA without altering the acidification, all GJs showed partial resistance to ischemic uncoupling. In calcium clamped condition, when pH_i_ was allowed to drop to the same extent, G_j_ of Cx40 reduced by 52±2% which is close to control value, suggesting that acidification is the major contributor in uncoupling. In the same condition, G_j_ of Cx43 and Cx45 reduced by 35±2% and 59±4% compared to 53±3% and 85±2% reduction in control (Figure 6 and Table 3). When pH_i_ change was restricted without affecting [Ca^2+^]_i_ rise, all Cxs showed only 26–29% reduction of G_j_ suggesting that acidosis indeed contributes more than [Ca^2+^]_i_ elevation in uncoupling. When both pH_i_ and [Ca^2+^]_i_ were maintained to the normal physiological levels, OGD had no effect on any of the GJs (Figure 7 and Table 3). It confirms that acidosis and [Ca^2+^]_i_ elevation are the primary causes of ischemic uncoupling of GJs. There are ample evidences suggesting that [Ca^2+^]_i_ works through CaM [21]. To check the involvement of CaM in ischemic uncoupling of GJ, we administrated anti-CaM antibody intra-cellularly through patch pipette. In case of Cx43, anti-caM antibody prevented uncoupling to a greater extent than BAPTA, suggesting its additional role, independent of calcium (Figure 6 and Table 3). CaM has been reported to bind to lens gap junction protein in calcium independent fashion [44]. In case of Cx45, buffering of [Ca^2+^]_i_ and inactivating CaM prevented the uncoupling to the similar extent, suggesting that [Ca^2+^]_i_ primarily worked through CaM in this case. Anti-CaM antibody had no effect on the uncoupling of Cx40. Unlike Cx43 and Cx45, Cx40 does not have putative CaM binding domain [45]. Therefore CaM promotes ischemic uncoupling of Cx43 and Cx45 but not in Cx40. It is not clear how CaM binding induces uncoupling [21], [45]. It has been proposed that CaM occludes channel mouth of Cx32 from cytosolic side inducing channel closure [20], [33]. This may be true also for Cx43 and Cx45. From the above discussion, it is apparent that lesser sensitivity to acidic pH makes Cx43 more resistant to OGD, compared to Cx45 and Cx40.

**Table 3:** % inhibition of junctional conductance (G_j_) by oxygen glucose deprivation (OGD).

Connexin	Control	[Ca^2+^]_i_ clamped	anti-CaM antibody	pH_i_ clamped	[Ca^2+^]_i_ and pH_i_ clamped
Cx45	85±2	59±4	54±2	27±1	5.9±0.2
Cx43	53±3	35±2	26±2	26±1	5.5±0.2
Cx40	64±1	52±2	65±2	29±2	7.2±0.4

OGD was performed for 5 minutes. [Ca^2+^]_i_ and pH_i_ were clamped by adding BAPTA (2 mM) and HEPES (80 mM) in pipette solution respectively. To restrict both [Ca^2+^]_i_ and pH_i_ change, 150 mM KPO_4_ was used in pipette.

To investigate the role of CT in ischemic uncoupling, we generated different CT truncated Cxs and chimeras. CT deletion mutant Cx43-*Δ*257 when exposed to OGD, uncoupling was attenuated significantly, suggesting its involvement in uncoupling (Figure 9). Earlier studies demonstrated that the intracellular loop, but not the CT of Cx43, is involved in calcium-CaM mediated reduction of G_j_ [45], [46]. Therefore, observed uncoupling in Cx43-*Δ*257 is possibly executed through calcium-CaM pathway. In the same line, OGD reduced the G_j_ of Cx43-*Δ*257 to the same extent of wild type Cx43, when pH_i_ was clamped in the latter. This suggests that CT of Cx43 is involved in acidosis mediated component of the uncoupling. This is consistent with the findings that CT-truncated Cx43 showed lesser sensitivity towards low pH, and resulted in increase of infarct size and arrhythmia due to acute coronary occlusion [36]. Upon tethering of CT of Cx43 to the truncated Cx40 and Cx45, not only did it improve their expression (Figure 8), but also the chimeras showed moderate sensitivity to ischemia (Figure 10). It was not possible to assess directly the contribution of CT of Cx40 and Cx45 in uncoupling, due to the low expression of truncated mutants. When CT of Cx40 was tethered to Cx43-*Δ*257, the sensitivity of the chimera to OGD did not improve much, suggesting its minor role in ischemic uncoupling (Figure 10). Cx45 chimera containing CT of Cx40 showed remarkably improved expression (Figure 8). However, OGD reduced the G_j_ of Cx45-C40 only by 38±1%, compared to 85±2% for Cx45 and 64±1% for Cx40. OGD mediated uncoupling of Cx40 is mainly due to acidosis and calcium-CaM had minimum contribution in it. If CT of Cx40 is involved in the reduction of G_j_, more robust uncoupling of Cx45-C40 would have been observed. Therefore CT of Cx40 possibly does not participate in ischemic uncoupling. We could not assess the role of CT of Cx45 in ischemic uncoupling as tethering of it with Cx43 or Cx40 did not yield a functional construct.

Apart from their role in ischemic uncoupling, we observed that the CT determines the characteristics of junctional plaque (Figure 8). In concurrence with previous report [47], removal of CT increased the plaque diameter made of Cx43. The G_j_ also improved (Table 1). However we could not detect junctional plaque in case of CT truncated Cx45 and Cx40. They also exhibited very little G_j_, possibly due to removal of phosphorylation and protein-protein interaction sites. Attachment of CT of Cx43 to truncated Cx40 and Cx45 enabled them to form junctional plaque with some altered properties. Plaque diameter of Cx40-C43 was smaller than Cx40 and appeared punctuated but the G_j_ of both were comparable. Intriguingly, tethering CT of Cx43 to truncated Cx45 resulted in bigger plaque than wild type Cx45 and increased G_j_. Similarly Cx45, when tethered to CT of Cx40, it formed distinct plaque and functional GJ. It suggests that CT of Cx43 is an essential component required for the expression and formation of junctional plaque and it can complement CT of Cx40 and Cx45. However it is not required for the formation of its own junctional plaque. CT of Cx40 is necessary for its expression and plaque formation; it can also complement CT of Cx45. In contrary, CT of Cx45 does not complement Cx40 or Cx43.

In accordance with previous reports, CT domain showed its involvement in voltage gating [37], [38], [42]. Truncation of CT reduced G_min_ and changed slope factor in case of Cx43 and Cx40. Following voltage step, I_j_ of CT-truncated Cx43 and Cx40 decayed mono-exponentially compared to biphasic decay of the corresponding wild type. Relaxation kinetics became slower for Cx43-*Δ*257, while it accelerated for Cx40-*Δ*249, as reported earlier [37], [38]. But truncation of the CT of Cx45 did not affect voltage dependent gating or relaxation kinetics (Figure S2). The only change observed was reduced surface expression and junctional plaque formation, resulting in decreased G_j_.

## Conclusions

This work figures out the molecular mechanisms that uncouple GJ channels formed of Cx43, Cx40 and Cx45 during ischemic condition. Ischemia was simulated by depriving oxygen and glucose. OGD caused the reduction of G_j_ of all Cxs significantly. Cx45 was most sensitive, while Cx43 showed maximum resistant to OGD. Elevated [Ca^2+^]_i_ and acidic pH_i_ were the primary causes, as uncoupling was prevented completely by restricting their change. Acidosis has greater contribution than elevated calcium in reducing the G_j_, particularly in the uncoupling of Cx40. Calcium worked through CaM, though calcium independent role of CaM in the uncoupling of Cx43 cannot be ruled out. Further, CT of Cx43 played significant role in ischemic uncoupling. Tethering of it to the CT-truncated Cx45 and Cx40 enabled them to respond to OGD, same like Cx43. CT also played significant role in determining plaque diameter, voltage dependent gating and current relaxation kinetics with the exception of Cx45-CT domain. Taken together, this study provides an explanation for the comprehensive mechanism of the ischemic uncoupling of cardiac gap junctions.

## Supporting Information

Figure S1 **V_j_ sensitivity of wild type and eGFP tagged Cx43 and Cx45.** **A**. Tagging of eGFP to the C-terminus of Cx43 decreases its voltage sensitivity. The G_j(ss)_-V_j_ plot shifted towards higher voltage. **B**. Voltage sensitivity of Cx45 did not change after tagging eGFP. G_j(ss)_-V_j_ plot of Cx45-eGFP is indistinguishable from that of wild type Cx45.
(TIF)

Figure S2 **Desensitization kinetics of wild type and CT-truncated connexin containing gap junctions.** **AI and AII**, representative I_j_ traces of Cx43 and Cx43-*Δ*257. V_j_ was stepped to 100 mV. I_j_ decayed mono-exponentially and bi-exponentially for truncated Cx43 and wild type Cx43 respectively. Predicted best fittings are presented with solid line. **AIII**, merged fitted curves of AI and AII. Corresponding τ values are indicated with arrows. **B**, relaxation kinetics of Cx40 and Cx40-*Δ*249. Figures are presented similar to fig. A. Current decay of Cx40 and Cx40-*Δ*249 are best fitted with double and single exponentially. **C**, voltage relaxation of Cx45 and Cx45-*Δ*272. Both Cx45 and truncated Cx45 showed double exponential decay of I_j_.
(TIF)

Table S1 Primers used for generating truncated C-terminal domain mutants.
(DOCX)

Table S2 Primers used for generating C-terminal chimeric mutants.
(DOCX)
