# Supplementary figures and images for: Contribution of Intracellular Calcium and pH in Ischemic Uncoupling of Cardiac Gap Junction Channels Formed of Connexins 43, 40, and 45: A Critical Function of C-Terminal Domain
Source: PLoS One. 2013 Mar 25;8(3):e60506. doi: 10.1371/journal.pone.0060506 (PMC3607587; doi:10.1371/journal.pone.0060506)

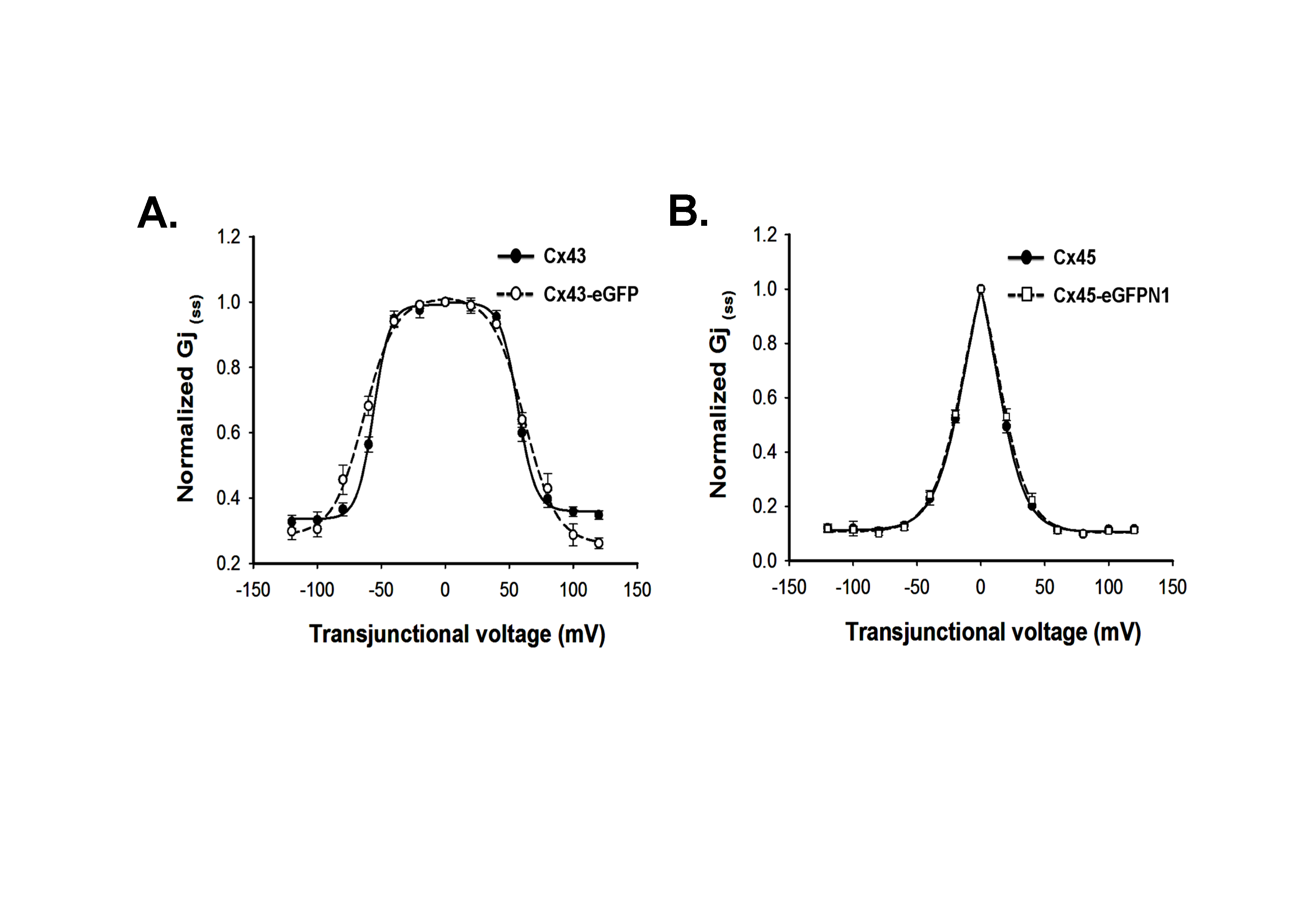

Supplement: Figure S1 — Vj sensitivity of wild type and eGFP tagged Cx43 and Cx45. A. Tagging of eGFP to the C-terminus of Cx43 decreases its voltage sensitivity. The Gj(ss)-Vj plot shifted towards higher voltage. B. Voltage sensitivity of Cx45 did not change after tagging eGFP. Gj(ss)-Vj plot of Cx45-eGFP is indistinguishable from that of wild type Cx45. (TIF) [file pone.0060506.s001.tif]

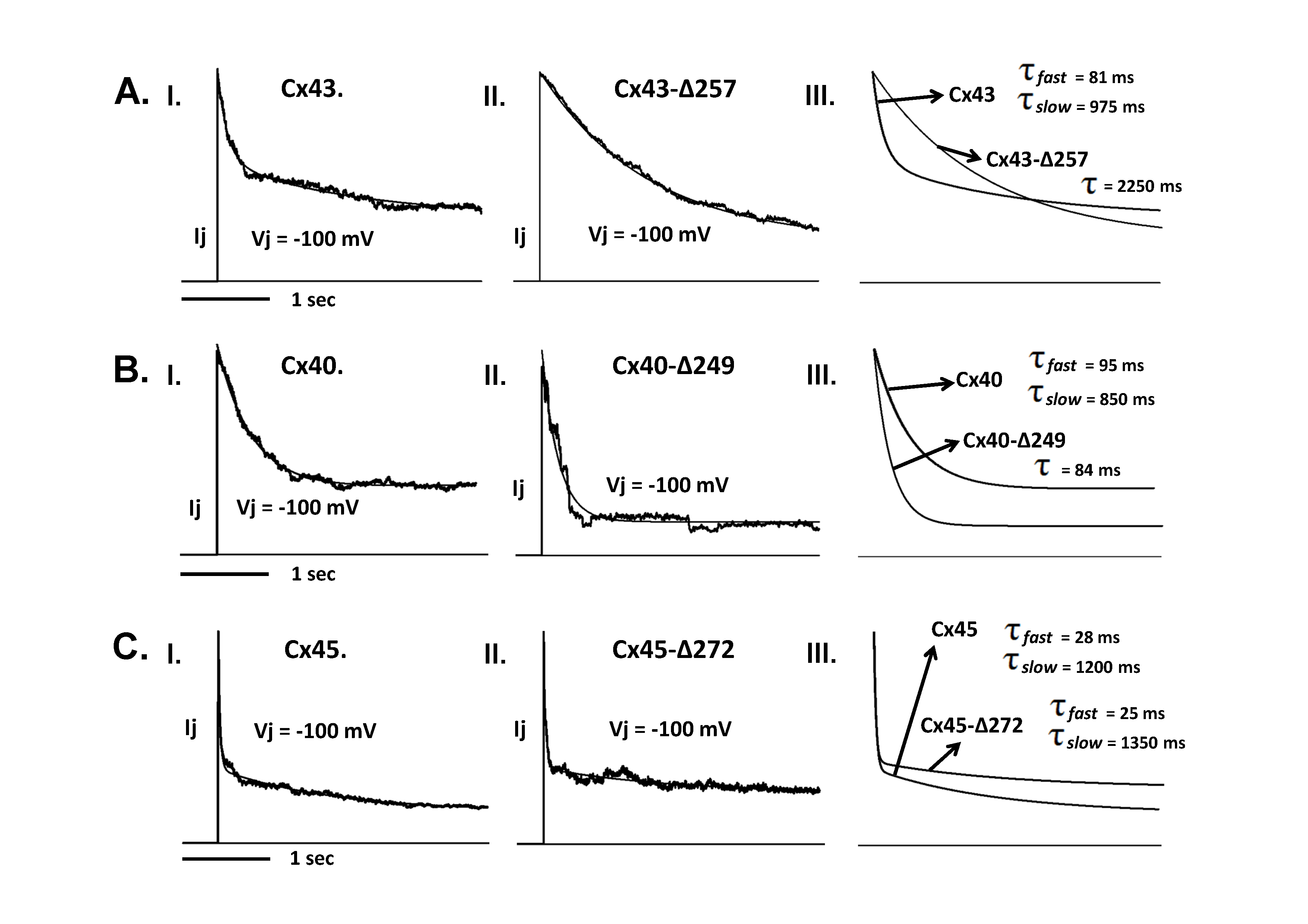

Supplement: Figure S2 — Desensitization kinetics of wild type and CT-truncated connexin containing gap junctions. AI and AII, representative Ij traces of Cx43 and Cx43-Δ257. Vj was stepped to 100 mV. Ij decayed mono-exponentially and bi-exponentially for truncated Cx43 and wild type Cx43 respectively. Predicted best fittings are presented with solid line. AIII, merged fitted curves of AI and AII. Corresponding τ values are indicated with arrows. B, relaxation kinetics of Cx40 and Cx40-Δ249. Figures are presented similar to fig. A. Current decay of Cx40 and Cx40-Δ249 are best fitted with double and single exponentially. C, voltage relaxation of Cx45 and Cx45-Δ272. Both Cx45 and truncated Cx45 showed double exponential decay of Ij. (TIF) [file pone.0060506.s002.tif]
