# Supplementary material for: Contribution of Intracellular Calcium and pH in Ischemic Uncoupling of Cardiac Gap Junction Channels Formed of Connexins 43, 40, and 45: A Critical Function of C-Terminal Domain
Source: PLoS One. 2013 Mar 25;8(3):e60506. doi: 10.1371/journal.pone.0060506 (PMC3607587; doi:10.1371/journal.pone.0060506)
Supplement: Table S1 — Primers used for generating truncated C-terminal domain mutants. (DOCX) [file pone.0060506.s003.docx]

**Table S1. Primers used for generating truncated C-terminal domain mutants:**

| Connexin | Primers used |
| --- | --- |
| Cx43-∆257 | For : 5' GCCCACTGAGCCCATAAAAAGACTGCGGATC 3'  Rev: 5' GATCCGCAGTCTTTTTATGGGCTCAGTGGGC 3' |
| Cx40-∆249 | For : 5' GTGGACAAGCACTAGCTGCCTGGCC 3'  Rev: 5' GGCCAGGCAGCTAGTGCTTGTCCAC 3' |
| Cx45-∆272 | For : 5' GGAGGGAACTTGATGATTAGGGTGCTTATAATTATCC 3'  Rev: 5' GGATAATTATAAGCACCCTAATCATCAAGTTCCCTCC 3' |

For: forward primer; Rev: reverse primer
