# Supplementary material for: Contribution of Intracellular Calcium and pH in Ischemic Uncoupling of Cardiac Gap Junction Channels Formed of Connexins 43, 40, and 45: A Critical Function of C-Terminal Domain
Source: PLoS One. 2013 Mar 25;8(3):e60506. doi: 10.1371/journal.pone.0060506 (PMC3607587; doi:10.1371/journal.pone.0060506)
Supplement: Table S2 — Primers used for generating C-terminal chimeric mutants. (DOCX) [file pone.0060506.s004.docx]

**Table S2. Primers used for generating C-terminal chimeric mutants:**

| Connexin | Primers used |
| --- | --- |
| Cx43-C40 | For : 5´-ACCACCGGCCCACTGAGCCCACAGCTGCCTGGCCCTC-3´  Rev: 5´-AGAGGGGCGGATCCGCTCACACTGACAGGTCATCTGAC-3´ |
| Cx43-C45 | For : 5´-ACCACCGGCCCACTGAGCCCACCGGGTGCTTATAATTATC-3´  Rev: 5´-AGAGGGGCGGATCCGCTTAAATCCAGACGGAGGTCTTC-3´ |
| Cx40-C43 | For : 5´-GGCAGGGTGTGGACAAGCACTCCAAAGACTGCGGATCTC-3´  Rev: 5´-TGGCCCTCCCTAAAAGGAGGATTAAATCTCCAGGTCATC-3´ |
| Cx40-C45 | For : 5´-GGCAGGGTGTGGACAAGCACCCGGGTGCTTATAATTATC-3´  Rev: 5´-TGGCCCTCCCTAAAAGGAGGATTAAATCCAGACGGAGG-3´ |
| Cx45-C43 | For : 5´-AGGAGGGAACTTGATGATTCCAAAGACTGCGGATCTC-3´  Rev: 5´-CGGATCCCGGGCCCCTTAAATCTCCAGGTCATCAGGCCG-3´ |
| Cx45-C40 | For : 5´-AGGAGGGAACTTGATGATCAGCTGCCTGGCCCTCCCAC-3´  Rev: 5´-CGGATCCCGGGCCCCTCACACTGACAGGTCATCTG-3´ |

For: forward primer; Rev: reverse primer
